# Benefits of Microwave-Assisted Heat Treatment for Sintered Diopside Glass-Ceramics

**DOI:** 10.3390/ma18020421

**Published:** 2025-01-17

**Authors:** Alexander Karamanov, Elena Colombini, Dario Ferrante, Ivan Georgiev, Miryana Raykovska, Emilia Karamanova, Stela Atanasova, Paolo Veronesi, Cristina Leonelli

**Affiliations:** 1 Institute of Physical Chemistry, Bulgarian Academy of Sciences, Acad. G. Bonchev Str., Bl. 11, 1113 Sofia, Bulgaria; ekarama@ipc.bas.bg (E.K.); statanasova@ipc.bas.bg (S.A.); 2 Department of Engineering “Enzo Ferrari”, University of Modena and Reggio Emilia, Via P. Vivarelli 10, 41125 Modena, Italy; elena.colombini@unimore.it (E.C.); 178830@studenti.unimore.it (D.F.); paolo.veronesi@unimore.it (P.V.); cristina.leonelli@unimore.it (C.L.); 3 Institute for Information and Communication Technologies, Bulgarian Academy of Sciences, Acad. G. 10 Bonchev Str., Bl. 2, 1113 Sofia, Bulgaria; ivan.georgiev@parallel.bas.bg (I.G.); mirianaraykovska@gmail.com (M.R.)

**Keywords:** sintering, glass-ceramics, diopside, microwave heating, tomography

## Abstract

Sinter-crystallization is a specific method of producing glass-ceramics that allows the manufacture of complexly shaped products, composites and solder. However, it usually is limited when the glass powders used are characterized by a high crystallization trend. This study proposes a new opportunity to improve the sinter-crystallization and demonstrates the benefits of microwave processing using diopside (CaMg(Si_2_O_6_)) glass-ceramics with an enhanced crystallinity of ~70%. The advantages of microwave processing are shown by comparing the results obtained with scanning electron microscopy, X-ray computed tomography and gas pycnometry for two glass-ceramic specimens. The first sample is obtained in the heat resistant furnace of an optical dilatometer, while the second is obtained by heating it with high-power microwave irradiation at 2.45 GHz, 1kW. Intense crystallization was observed in the sample sintered in an electric furnace, which blocked the sintering process and resulted in significant open porosity (7.1%). In addition, closed pores caused by the crystallization are observed in the centers of the sintered particles (5.2%). At the same time, the overall porosity of the microwave-sintered glass-ceramic is reduced by about two times, and the open porosity is practically eliminated (0.5%). In this sample, together with the crystallization-induced pores, some residual closed spherical pores, typical for a well-sintered sample, are also observed.

## 1. Introduction

Sinter-crystallization is considered an alternative technique for the manufacture of glass-ceramics, which enlarges the application range of these materials [1,2]. It is very helpful when samples with a complex shape, obtained by 3D printing [3], injection molding [4] or other advanced methods [5], are used.

During sinter-crystallization, two competing processes, densification and crystallization, which require comparable atomic mobility, take place in similar temperature ranges. Inevitably, these phenomena interact with each other, accounting for the various problems to be solved. The main difficulty arises when the crystallization increases the effective viscosity so rapidly that the densification remains uncomplicated.

If the crystallization tendency of the parent glass is low, it is possible to complete the densification before the actual start of crystallization so that the process can be explained by the traditional models of glass sintering [6,7,8]. In this case, in order to avoid the deformation, the final samples can be obtained at low heating rates and after a prolonged crystallization step; unfortunately, their crystallinity is lower.

However, in most of the sintered glass-ceramics, the crystallization starts before the end of sintering and can therefore inhibit it. In this case, the initial stages of the sinter-crystallization can also be explained by the viscous flow models, while then the sintering rate progressively starts to decrease due to the crystallization [8,9,10]. Often, after the formation of a critical amount of solid phase, the densification is completely blocked, and the final structure depends on the degree of densification achieved prior to this inhibition. In addition, during the subsequent crystallization, extra porosity may be created due to the density difference between the parent glass and the newly formed crystal phase. When the main crystal phase is pyroxene, this difference is extremely high (at about 16 vol%), and the crystallization-induced porosity might reach 6–7% [11,12,13].

In general, the higher the crystallinity, the faster the phase formation, resulting in lower sintering ability. At the same time, it is supposed that the higher crystallinity of glass-ceramics favors their mechanical characteristics. Therefore, the production of well-sintered glass-ceramics with enhanced crystallinity can be considered a technological and scientific challenge. Well-sintered glass-ceramics with high crystallinity can be obtained by using hot pressing or hot isostatic pressing [5]. However, they are cost-effective processes that can be used for high-value-added glass-ceramic products [14].

Alternatively, very fine glass particles are typically used. But, in this case, the milling procedure is expensive, and, in addition, the large surface area of the glass particles can cause some foaming [15,16,17]. Also, due to the higher initial porosity of the “green” samples, the firing shrinkage will be greater, which is a prerequisite for some technological problems.

Sometimes, the sintering can be improved by using higher heating rates [18,19,20] or secondary sintering close to liquidus temperatures [19,21]. Unfortunately, especially for larger samples, the application of these methods is also limited due to problems with the heat transfer between the surface and the volume. The difficulties with the temperature gradient, created at very high heating rates, can be overcome by microwave-assisted heat treatment. In fact, it offers the possibility of very rapid heating combined with the formation of homogeneous ceramic [22] and glass-ceramic [23,24,25] structures and improved properties. In addition, the microwave-assisted sintering has a lower production cost than the traditional heat treatment [26].

The aim of this study is to demonstrate the advantage of microwave heating in the sinter-crystallization of glass-ceramics with high crystallinity. A model diopside (CaMg(Si_2_O_6_)) glass-ceramic that forms at a ~70 wt% crystal phase was used because of its high tendency to crystallize via surface crystallization and the large density difference between the crystalline and glassy states of diopside (3.27 and 2.75 g/cm^3^, respectively [11]).

## 2. Experimental Section

In the present work, a glass with the following theoretical composition was used: SiO_2_—52, CaO—22, MgO—22, B_2_O_3_—2 and Na_2_O—2 (mol %). Its parent batch was prepared by mixing silica sand (SiO_2_ > 99.5%) and technically pure H_3_BO_3_, CaCO_3_, MgCO_3_ and Na_2_CO_3_ (Merck, Burlington, MA, USA). The melting was carried out in a Kanthal^®^ Super electric furnace (Georgiev Ltd., Sofia, Bulgaria) at 1500 °C for 2 h using a 200 mL corundum crucible. The melt was quenched in water, and the resulting frit (about 250 g) was dry-ground in a ball mill (Fritsch—Idar-Oberstein, Germany) and then sieved to obtain fraction between 75 and 140 µm (CISA sieve—Barcelona, Spain).

The intensive crystallization of glass powders was demonstrated by DTA (PerkinElmer—Diamond, Shelton, CT, USA) at 10 °C/min.

The sintering process was studied with a combined hot-stage microscope and horizontal contactless optical dilatometer apparatus (Misura HSML ODLT 1400, Expert System Solutions—Modena, Italy) equipped with a Pt-Rh furnace using the same heating rate.

“Green” samples (pellets) with size of 50/5/4 mm^3^ were prepared by mixing the glass powders with 7 wt% polyvinyl alcohol (PVA) solution and pressing at 40 MPa (uniaxial hydraulic press, Nannetti—Faenza, Italy). A preliminary burn-out step of 30 min at 270 °C was carried out before the sintering experiments. The samples obtained by isothermal dilatometric experiment, labeled GC-OD, were then used to evaluate their porosity, crystallinity, structure and morphology.

Alternatively, other samples were heat-treated non-isothermally in a microwave-assisted furnace with a rectangular TE10 single-mode applicator fed by a magnetron generator at 2450 MHz with an output power level of 100 to 3000 W (MKS-Alter, Reggio Emilia, Italy). As microwave absorbers at lower temperatures, SiC pads were used. The temperature was monitored with an optical pyrometer (IKS-T14-09, Sitel Control Srl, Milan, Italy). In practice, depending on whether the sample starts to deform or not, it is claimed that the “real” temperature reached is below or above the deformation range of 1150–1200 °C. Considering that the total heating time was between 20 and 25 min, it can be assumed that an average heating rate was 50–60 °C/min.

**Figure 1:**
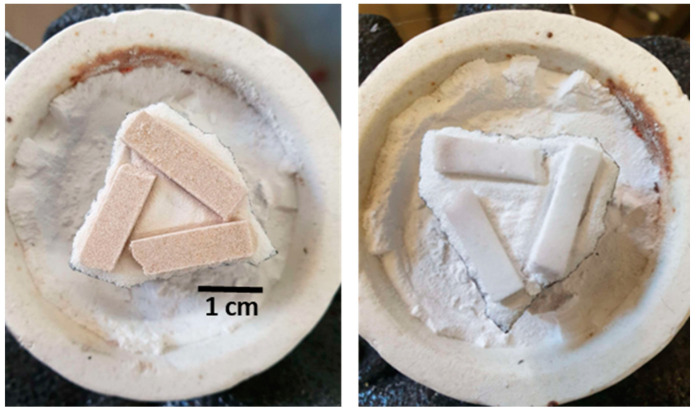
GC-MW samples before (**left**) and after (**right**) microwave treatment.

Figure 1 shows the photos of samples before and after microwave treatment. Some of the non-deformed samples thus obtained, labeled as GC-MW, were selected and used together with GC-OD in the subsequent experiments.

The phase analysis of both samples was studied by XRD (Philips—PW1830—Almelo, The Nederland) using powdered glass-ceramic samples.

In order to obtain information on the degree of sintering achieved for both samples, the porosity was evaluated using traditional methods. The apparent (*ρ*_a_), skeletal (*ρ*_s_) and absolute (*ρ*_as_) densities and the water absorption (*WA*) were determined. *ρ*_a_ was estimated using a precision micrometer and balance, while *ρ*_s_ and *ρ*_as_ were evaluated using a gas (Ar) pycnometer (AccuPyc 1330, Micromeritics—Norcross, GA, USA) before and after crushing and milling of the samples below 26 μm, respectively. *WA* was measured after boiling in distilled water for 3 h. The results were used to calculate closed *P_C_* and open *P_O_* porosities by means of the following relationships:

$$P_{C}=100\;\times \;\frac{\rho_{\mathrm{as}}-\rho_{s}}{\rho_{\mathrm{as}}}\;[\mathrm{vol}\%];\;P_{O}=WA\;\times \;\rho_{a}\;[\mathrm{vol}\%]$$

Crystallinity was estimated by the difference between the absolute density of parent glass (2.70 g/cm^3^) and those of both glass-ceramics [12].

The microstructure and morphology of the surfaces and polished fractures of the two final glass-ceramics were investigated using a field-emission scanning electron microscope (JEOL IT800SHL—Tokio, Japan) with both secondary and backscattered electron detectors placed in-chamber and in-lens microscope columns.

In addition, the overall microstructures were studied by X-ray computed tomography. The samples were scanned using a micro-computed tomography system (Nikon Metrology—Tring, UK), providing a resolution of 4 μm with a continuous 360° rotation, 180 kV/200 µA. A total of 2880 images were acquired during each scan with an exposure time of 1000 s. Reconstruction of the tomographic data was carried out using NikonMetrology’s CT Pro-3D software (Nikon Metrology, Hertfordshire, UK), and porosity analysis was subsequently performed using VG STUDIO MAX version 2023.4. Following automatic surface determination for each of the two specimens, regions of interest with uniform dimensions were selected to standardize the analysis. Porosity measurements were then performed within these consistent volumes.

## 3. Results and Discussion

**Figure 2:**
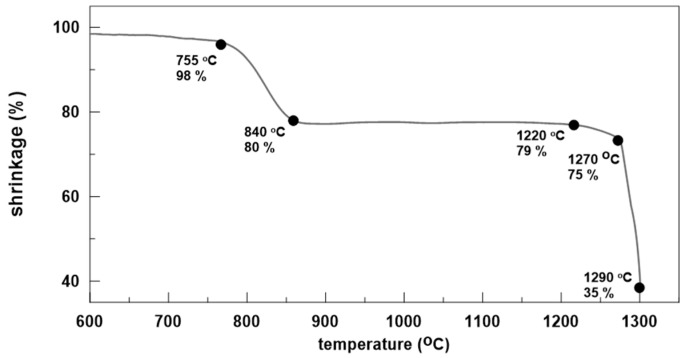
HSM plot of the studied glass powders.

The sintering behavior of glass powders was first studied with HSM. The resulting plot is shown in Figure 2, while the temperatures corresponding to the “fixed” points automatically evaluated by the HSM equipment and the associated viscosities [27] are summarized in Table 1. From Figure 2, it can be seen that the densification starts at about 755 °C and finishes at about 840 °C, reaching a shrinkage of ~20%. No sintering or deformation is then observed up to 1200–1210 °C. With further increase in the temperature, some secondary densification occurs, and at 1270 °C, the shrinkage increases to ~25%. This behavior shows that above 840 °C, an intensive crystallization process, which completely inhibits the sintering, occurs.

After 1270 °C, due to the rapid melting of the formed crystal phase, deformation starts, followed by a drastic decrease in the apparent viscosity and melting. As a result, the difference between the softening and melting temperatures is only about 20 °C.

**Figure 3:**
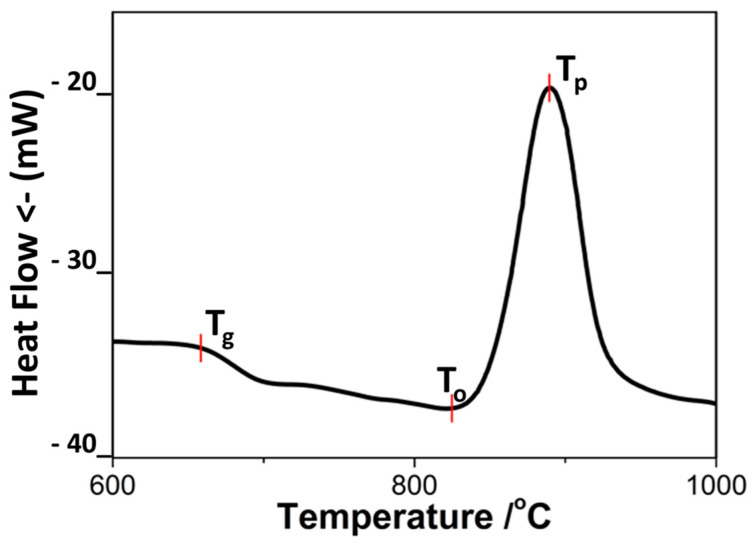
DTA result of the studied glass powders.

Figure 3 shows the corresponding DTA plot confirming the high crystallization trend. The glass transition temperature (T_g_) occurs at 665 °C, while the first crystallization onset (T_o_) and the crystallization peak temperature (T_p_) are at around 825 and 890 °C, respectively. The crystallization exo-thermic effect is very intense, and the difference ΔT between T_g_ and T_p_ is only ~230 °C.

This temperature difference is widely used to compare the crystallization trends in glasses with similar compositions [28]. The higher the crystallization ability, the closer the crystallization peak is to one of the glass transitions, which means that the phase formation is carried out at higher viscosity. This simple relationship explains why the use of glasses with superior crystallization ability can lead to problems with densification.

Conversely, at a lower crystallization tendency, the phase formation is slower, and the DTA peak occurs at higher temperatures (i.e., at lower viscosity). In fact, in previous studies with similar diopside model compositions, having lower CaO and MgO amounts (with 1–2 mol%) and using comparable fraction size and heating rates, ΔT was higher by 20–30 °C [11,29]. This difference indicates that the phase formation in these compositions takes place at three to four times lower viscosities; as a result, no significant problems with the sintering were observed. The obtained samples were characterized by 8–10% porosity and crystallinity below 60% [11]. However, their bending strength (130–140 MPa) and Young’s modulus (105–110 GPa) can be considered promising starting values.

After some preliminary tests, a heat treatment at 10 °C/min up to 830 °C (i.e., near the onset of the first crystallization DTA peak) followed by a two-hour isothermal step was chosen as an appropriate thermal cycle for the synthesis of GC-OD. No significant improvement in the achieved degree of sintering was observed by applying higher holding temperatures or by increasing the heating rate up to 15–20 °C/min.

**Figure 4:**
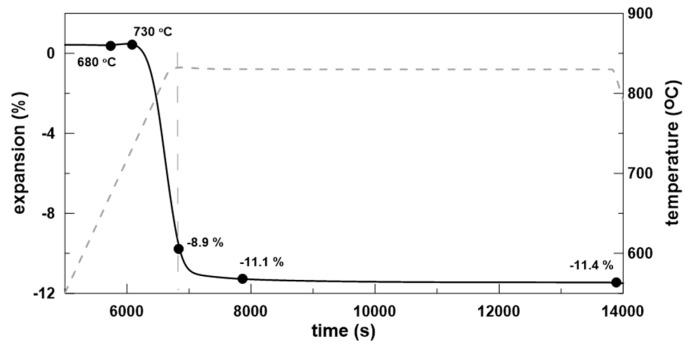
Dilatometric sintering curve for GC-OD sample.

The corresponding dilatometric sintering plot (solid line) and thermal regime (dashed line) are presented in Figure 4 and shows T_g_ and dilatometric softening point, T_s_, at about 680 and 730 °C, respectively. These values, which are in good agreement with the viscosity points estimated by HSM and DTA, are also included in Table 1.

As demonstrated in Figure 4, the densification process is remarkably rapid, occurring predominantly during the heating stage. The shrinkage observed during the holding step is minimal, with a maximum of 2% recorded within the initial 10–15 min. The ongoing crystallization is not related to volume changes of the sample. It can be assumed that the densification process, accompanied by some crystallization shrinkage, is completed at the beginning of the holding step; then, due to the intensive surface crystallization, the system rapidly becomes rigid, and the volume variations are completely inhibited. This means that the phase formation that continues after this point is not related to crystallization shrinkage but to the formation of crystallization-induced pores [11,12,13].

The apparent, skeletal and absolute densities and the water absorption values for the GC-OD samples thus synthesized, together with the corresponding values for the GC-MW samples obtained by microwave treatment, are summarized in Table 2, together with the resulting values for open and closed porosities.

The density measurements show significant differences. In GC-OD, an evident open porosity is presented, confirming that the sintering process is not complete, whereas in GC-MW, the open porosity is practically eliminated. At the same time, the differences in the skeletal and absolute densities are not so significant, and the closed porosity in the sample obtained by microwave treatment is higher at less than 2%.

The variance between the absolute density of parent glass and the two glass-ceramics corresponds to a high crystallinity of about 68–72%, as calculated from the density ratio reported in reference [11]. This is a key finding, highlighting that despite the significant improvement of the sintering, the crystallinity of GC-MW remains elevated.

**Figure 5:**
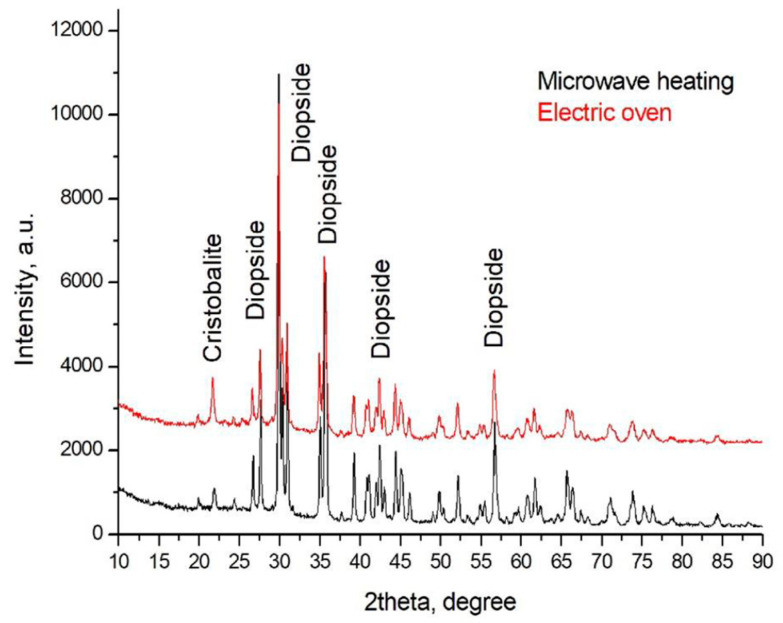
XRD pattern of both samples.

In order to confirm the significant presence of diopside in the sintered samples, the X-ray diffraction on powdered specimens was collected (Figure 5). The XRD patterns of both glass-ceramics are shown in Figure 5 and confirm the formation of similar high amounts of diopside in both samples.

The differences in the structure were clarified with SEM and CT observations.

**Figure 6:**
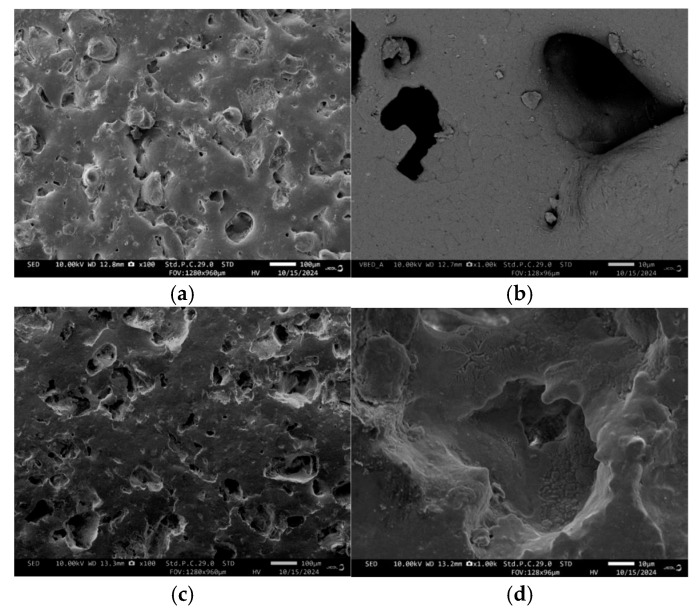
SEM images of the surfaces of GC-OD (**a**,**b**) and GC-MW (**c**,**d**).

**Figure 7:**
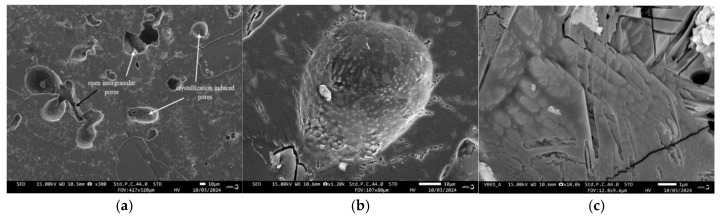
SEM images of different pore morphologies observed on the polished fractures of GC-OD (**a**), typical crystallization induced pore (**b**) and diopside crystals (**c**).

**Figure 8:**
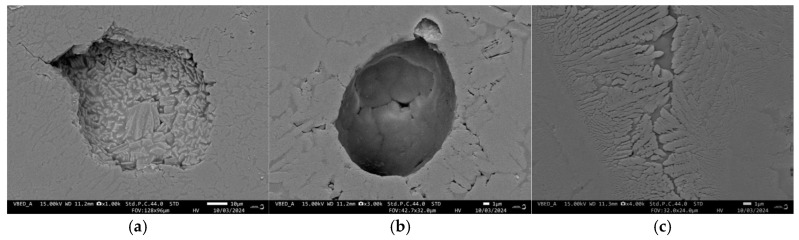
SEM images of different morphologies observed on the polished fracture of GC-MW: crystallization induced pore (**a**), smooth residual pore (**b**) and diopside crystals (**c**).

Typical SEM images of the surfaces of both samples are shown in Figure 6, while their polished fractures are elucidated in Figure 7 and Figure 8.

The surface of the GC-OD sample (Figure 6a) shows some distinct particles, together with various open pores with irregular shapes (Figure 6b), while the surface of the GC-MW sample (Figure 6c) shows a smoother surface and almost spherical “shallow” closed pores (Figure 6d).

The difference in the degree of sintering is also confirmed by the observations of the cross-sections of samples. The GC-OD images show that the largest pores are open, with a complex shape and sizes mostly between 90 and 130 µm (Figure 7a). Some interconnected “groups” of pores, reaching 250–300 μm, are also identified. Other typical pores are the closed intragranular crystallization-induced pores, which are formed in the centers of each grain and are characterized by spherical shape and size between 30 and 60 µm (Figure 7b). It can be noted that the residual intergranular pores are characterized by a smooth surface, whereas the crystallization pores have the typical polycrystalline „dentate“ surface due to the crystal growth that ends in the central void of the grains.

In contrast, the GC-MW sample shows significantly lower porosity and practically no large open pores. The pores are mainly spherical, with sizes between 20 and 90 µm. The majority of the pores, as shown in Figure 8a, are somewhat similar to the crystallization-induced pores in GC-OD. However, another part of the pores (see Figure 8b) is characterized by a smooth surface, which is typical for residual intergranular pores in a well-sintered glassy material.

**Figure 9:**
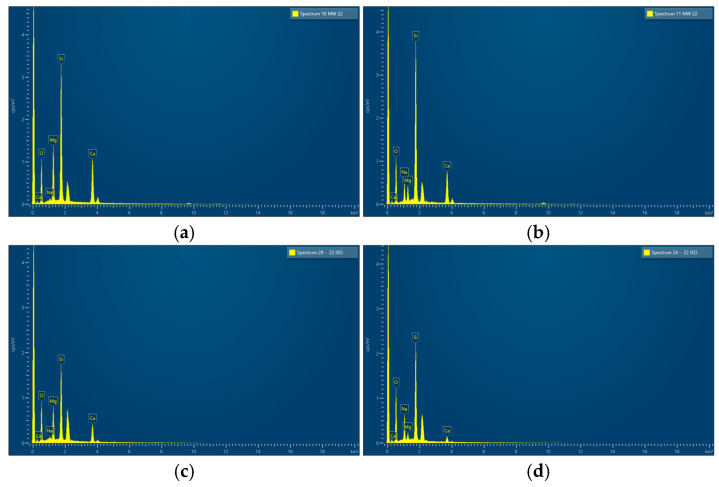
EDS spectra of crystals ((**a**) GC-MW and (**c**) GC-OD) and residual amorphous phases ((**b**) GC-MW and (**d**) GC-OD).

The characteristic crystalline habitus of diopside dendritic crystals growing from the surface to the interior of the glass particle is shown in Figure 7c and Figure 8c for GC-OD and GC-MW, respectively. Typical EDS spectra of the crystals and residual amorphous phases in both samples are presented in Figure 9. These results clearly demonstrate the higher amounts of CaO and MgO in the diopside and the increased concentrations of Na_2_O in the residual glassy phase.

Due to the high crystallization tendency and the relatively coarse fraction size of the powder used, the densification in GC-OD stops after the formation of a certain surface crystallized layer in the sintered particles, forming a structure with some open pores. Crystallization-induced pores then begin to form in the centers of the grains, further increasing the overall porosity. As a result, both kinds of pores are well differentiated (Figure 7a).

On the contrary, during rapid microwave heating, the densification is almost complete before the formation of a critical amount of crystalline phase so that the open porosity is practically eliminated. It can be assumed that most of the closed porosity is also a consequence of the large density difference between the crystalline and amorphous structures of diopside. At the same time, the formation of spherical closed pores with a smooth surface, typical of a completed sintering process [30], could be explained by the assumption that the sintering process is accelerated so efficiently that full densification is achieved before the start of intensive crystallization.

Such a significant improvement in densification cannot be explained solely by the higher heating rate during microwave sintering. In fact, it is well known that microwaves are oscillating electromagnetic energy with frequencies in the range of 300 MHz to 300 GHz, with low-cost sources positioned at 2.45 GHz. Most of the microwave–matter interactions relevant to ceramic processing are due to electric field-induced polarization and reorientation phenomena, especially in the absence of magnetic properties [31]. In practice, the degree of conversion of electromagnetic energy into heat can be expected to depend on the permittivity of the ceramic material irradiated by the microwaves. In addition, when the sample immersed in the electromagnetic field is in the form of small particles, the electric field can be amplified at the highest curvature, leading us to believe that the role of the so-called “ponderomotive force” can also be considered [32]. This effect, which is active in the presence of an intense electromagnetic field, leads to enhanced surface mass transport mechanisms. This favorable idea needs to be clarified with additional experiments. If it is confirmed, a new possibility for successful synthesis of sintered glass-ceramics with enhanced crystallinity and improved properties could be realized.

**Figure 10:**
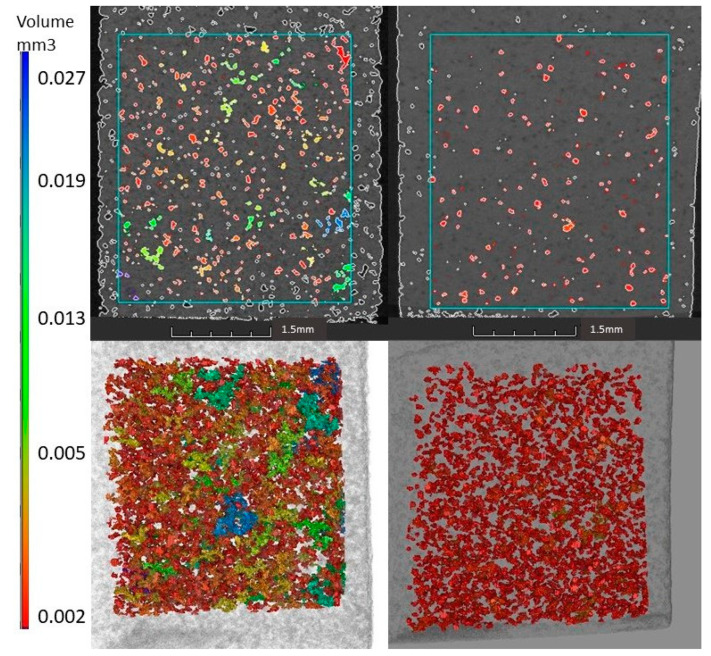
Two-dimensional slices (**top**) from the tomographic scan of the studied zones and three-dimensional renderings of the same regions (**bottom**). GC-OD sample (**right**) and GC-MW sample (**left**).

The SEM results were supplemented by the X-ray computed tomography studies summarized in Figure 10. The top panels show two-dimensional sections of the tomographic scan, elucidating the regions of interest with a color-coded visualization of porosity. The bottom panels show three-dimensional renderings of the same regions, with a 3D color-coded visualization of the porosity distribution within the selected volumes. This comparative analysis between the 2D and 3D views provides a comprehensive understanding of the spatial arrangement and density of pores within the scanned material.

In GC-MW, the porosity is clearly lower, and the voids are mainly near-spherical, while in GC-OD, larger pores with irregular shapes and groups of interconnected pores are observed. It is also evident that in GC-MW, the pores are isolated (i.e., closed), whereas in GC-OD, groups of interconnected pores of different sizes and shapes are observed. The 3D reconstruction shows that the pores are regularly distributed in the volume of both samples.

At the specified resolution and selected equivalent volumes, 1761 pores were identified in the GC-OD sample, compared to 1741 pores that were detected in the GC-MW sample. However, the measured total pore volumes were 1.43 mm^3^ and 0.64 mm^3^, corresponding to porosities of 8.0% and 3.6% for GC-OD and GC-MW, respectively.

Taking into account the resolution of the computed tomography method used, which does not provide satisfactory information for pores smaller than 8–10 µm, the porosity in both samples could be expected to be higher and comparable to the values obtained by gas pycnometry.

**Figure 11:**
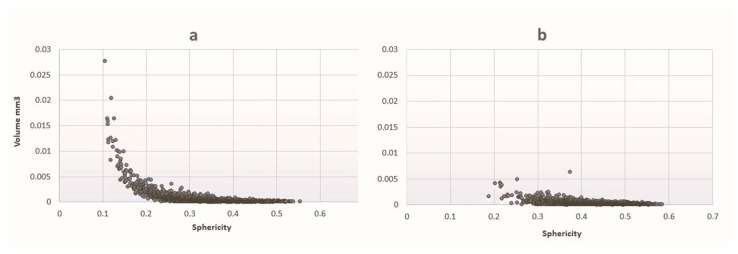
Graphical analysis of pore volume vs. sphericity for GC-OD (**a**) and GC-MW (**b**).

Figure 11 shows a graphical analysis of pore volume versus sphericity for both samples. This comparison allows an assessment of the differences in pore morphology and structural characteristics between the two specimens. It can be seen that the majority of pores in both samples are slightly spherical and have volumes below 0.003–0.005 cm^3^. However, larger pores with a volume exceeding 0.01–0.015 cm^3^ are observed only in the GC-OD sample. In addition, the sphericity of these pores decreases with the increase in their size, so they must be interpreted as interconnected open pores.

## 4. Conclusions

Preliminary results for the positive effect of microwave-assisted heat treatment on the sintering of a diopside glass-ceramic, forming a high amount of crystal phase, are reported. When traditional heat treatment is used, due to the high crystallization tendency of used glass, the sintering is inhibited, and about 7 vol% open porosity remains in the sample together with about 5 vol% closed porosity, which can be explained as crystallization induced. However, by applying a short microwave treatment, the total porosity is reduced by half without notable variation in the crystallinity. The open porosity is virtually eliminated, while the residual closed porosity of about 6.5 vol% again can be explained mainly by the formation of crystallization-induced pores.

## Figures and Tables

**Table 1:** Characteristic temperatures (°C) and viscosities (Pa·s), according to HSM test, DTA and optical dilatometry.

	Log η	Temperature
Tg—DTA	12.3	665
Tg—Dil	12.3	680
Ts—Dil	10.0	730
First Shrinkage (T_FS_)	9.0	755
Maximum Shrinkage (T_MS_)	7.2	840
Softening (T_S_)	5.1	1270
Flow (T_F_)	3.1	1290

**Table 2:** Apparent, skeletal and absolute densities and water absorption (WA) values, together with the corresponding open (P_o_) and closed (P_c_) porosity values. Instrument sensitivity is the error associated with these values.

	GC-OD	GC-MW
ρ_app_ (g/cm^3^)	2.55 ± 0.01	2.77 ± 0.01
ρ_sch_ (g/cm^3^)	2.88 ± 0.005	2.82 ± 0.005
ρ_abs_ (g/cm^3^)	3.05 ± 0.005	3.03 ± 0.005
WA (%)	2.8 ± 0.1	0.2 ± 0.1
P_o_ (%)	7.1 ± 0.3	0.5 ± 0.3
P_c_ (%)	5.2 ± 0.1	6.6 ± 0.1

## Data Availability

The original contributions presented in this study are included in the article. Further inquiries can be directed to the corresponding author.
